# Intelligent Health Monitoring in 6G Networks: Machine Learning-Enhanced VLC-Based Medical Body Sensor Networks

**DOI:** 10.3390/s25113280

**Published:** 2025-05-23

**Authors:** Bilal Antaki, Ahmed Hany Dalloul, Farshad Miramirkhani

**Affiliations:** Department of Electrical and Electronics Engineering, Isik University, 34980 Istanbul, Turkey; bilal.antaki@isikun.edu.tr (B.A.); 22elec5002@isik.edu.tr (A.H.D.)

**Keywords:** adaptive modulation, artificial intelligence (AI), channel modeling, channel parameter estimation, machine learning (ML), visible light communication (VLC)

## Abstract

Recent advances in Artificial Intelligence (AI)-driven wireless communication are driving the adoption of Sixth Generation (6G) technologies in crucial environments such as hospitals. Visible Light Communication (VLC) leverages existing lighting infrastructure to deliver high data rates while mitigating electromagnetic interference (EMI); however, patient movement induces fluctuating signal strength and dynamic channel conditions. In this paper, we present a novel integration of site-specific ray tracing and machine learning (ML) for VLC-enabled Medical Body Sensor Networks (MBSNs) channel modeling in distinct hospital settings. First, we introduce a Q-learning-based adaptive modulation scheme that meets target symbol error rates (SERs) in real time without prior environmental information. Second, we develop a Long Short-Term Memory (LSTM)-based estimator for path loss and Root Mean Square (RMS) delay spread under dynamic hospital conditions. To our knowledge, this is the first study combining ray-traced channel impulse response modeling (CIR) with ML techniques in hospital scenarios. The simulation results demonstrate that the Q-learning method consistently achieves SERs with a spectral efficiency (SE) lower than optimal near the threshold. Furthermore, LSTM estimation shows that D1 has the highest Root Mean Square Error (RMSE) for path loss (1.6797 dB) and RMS delay spread (1.0567 ns) in the Intensive Care Unit (ICU) ward, whereas D3 exhibits the highest RMSE for path loss (1.0652 dB) and RMS delay spread (0.7657 ns) in the Family-Type Patient Rooms (FTPRs) scenario, demonstrating high estimation accuracy under realistic conditions.

## 1. Introduction

The recent rapid development of wireless communication applications, especially those supported by Artificial Intelligence (AI), necessitates revolutionary advancements in communication technologies. While Fifth Generation (5G) systems are being deployed globally, industry and academia are exploring the potential of Sixth Generation (6G) systems [1]. Although 5G introduced substantial advancements, it still faces challenges related to reliability, latency, bandwidth, and data rate, which 6G aims to address. The 6G communications evolution introduces a major leap in wireless connectivity since it upgrades network capabilities with Ultra-Reliable Low-Latency Communications (URLLCs), Enhanced Mobile Broadband (eMBB), Massive Machine-Type Communications (mMTCs), and phenomenal terabit-per-second data speed communication, which opens the door for innovative services and applications. Major service comparisons for both 5G and 6G using various sets of key performance indicators (KPIs) are illustrated in Table 1. Moreover, 6G utilizes AI and machine learning (ML) [2,3] to simplify and optimize network management, dynamically allocate spectra, enhance security, and enable context-aware communication. This integration of ML ensures intelligent, adaptive networks that efficiently allocate resources, support autonomous systems, and deliver personalized communication experiences, making 6G a transformative leap in wireless technology [4,5]. To highlight the benefits of incorporating ML into wireless communication systems, the authors in [6] proposed a deep learning (DL) framework for modeling link-level Multiple-Input Multiple-Output (MIMO) channel scenarios. They further validated the model through cross-validation techniques and power analysis, demonstrating its reliability, effectiveness, and consistency for DL-based Channel State Information (CSI) feedback tasks.

**Figure 1:**
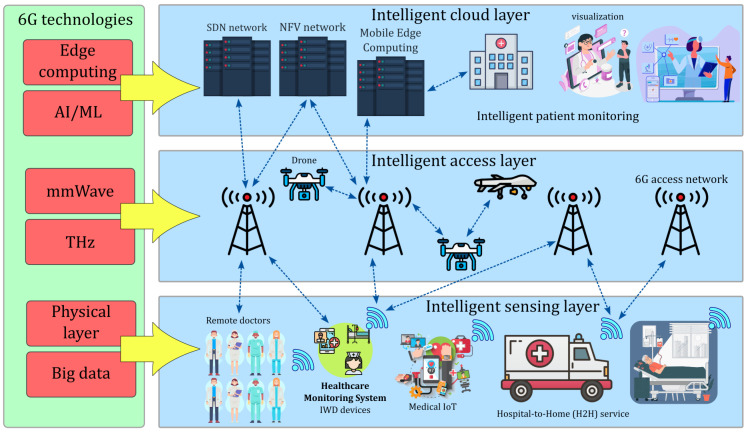
Healthcare network architecture within 6G.

A technology that shows promise for 6G and beyond is Optical Wireless Communication (OWC), which involves optical transmission in unguided media categorized by operating frequency [7]. OWC addresses spectrum shortages with ultra-high bandwidth, unregulated spectra, and high data rates. Furthermore, Visible Light Communication (VLC)—a subset of OWC that uses the visible light spectrum for indoor data transmission and positioning—optimizes traditional indoor applications and is, therefore, a promising candidate for the 6G communications landscape. In [8], a VLC-enabled ML proposed a unified physical-layer mixed carrier communication that elevates performance and localization. The results show superior optimization, with a 12–15 dB signal-to-noise ratio (SNR) gain at a target symbol error rate (SER) of $10^{-3}$, optimized up to 40% in spectral efficiency (SE). Moreover, integrating VLC within 6G networks addresses wireless connectivity challenges by presenting hybrid communication systems that take advantage of combining both Radio Frequency (RF) communication and VLC to deal with network problems within high electromagnetic interference (EMI) areas or dense urban environments. This hybrid communication takes advantage of VLC abilities, such as enhancing the security within line-of-sight environments for essential 6G applications like the Internet of Things (IoT) and healthcare environments [9], providing more enhanced data rates and reliability. 6G healthcare applications will support efficient home care and manage large patient volumes by utilizing different 6G technologies in a smart sensor layer, a smart access layer, and a smart cloud layer, as depicted in Figure 1 [10]. The figure illustrates the 6G healthcare network’s architecture, utilizing several key enabling technologies for 6G.

**Figure 2:**
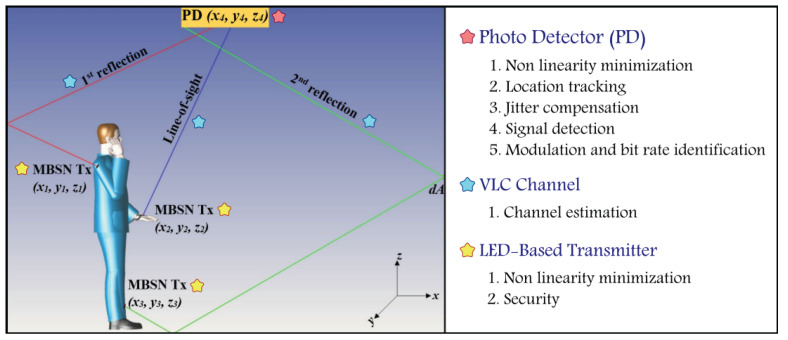
ML applications in a VLC-Based MBSN system.

Furthermore, utilizing VLC within 6G technology with Medical Body Sensor Networks (MBSNs) represents an extraordinary advancement in the realm of healthcare since VLC-based MBSN will enable seamless wireless communication between medical detectors and external devices. Healthcare environments such as hospitals and clinics show increasing demand and reliance on various technologies like Wireless Sensor Networks (WSNs), the Internet of Medical Things (IoMT), Telemedicine, and Biomedical Signal Processing, which employ real-time physiological parameters monitoring for patients that grant timely interventions and early detection of health deterioration. In order to demonstrate the capability of utilizing ML within WSN, the work in [11] highlights the growing role of ML in enhancing Wearable Health Monitoring (WHM) models. The study provides a comparison between conventional ML and emerging DL techniques, such as Sparse Coding autoencoders and Recurrent Neural Networks (RNNs), emphasizing their superior accuracy performance in processing time-series sensor-based data, the automatic extraction of features, activity recognition, and classification. Moreover, the authors of [12] proposed a deep learning-based WSN framework model for real-time healthcare monitoring and disease prediction. The model achieved a classification accuracy of 96%, with a loss rate of 0.08, outperforming traditional approaches by more than 5% in accuracy. Together, these studies reinforce the advantages of integrating ML into VLC-based WSN applications for improved performance and reliability in health monitoring. Integrating a VLC-based MBSN system plays a crucial role in the 6G ecosystem, particularly for applications such as electronic health (eHealth), indoor accuracy, underwater communication, and sensing systems. In these environments, VLC-based MBSNs enable precise localization and sensing technologies with a strong emphasis on supporting 6G massive IoT and URLLC, as shown in [13]. Additionally, the integration of AI/ML with VLC-based MBSNs addresses challenges like Light Emitting Diode (LED) nonlinearities, environmental effects, and security vulnerabilities. It improves position tracking, phase estimation, channel estimation, and modulation detection, as illustrated in Figure 2 [14]. This combination not only ensures efficient, high-throughput, and reliable communication but also supports the broader 6G goals of optimized resource allocation, security, and intelligent connectivity.

Different essential requirements are needed when designing multiple hospital setting scenarios, such as the Intensive Care Unit (ICU), semi-private patient rooms, Family-Type Patient Rooms (FTPRs), and clinics. In healthcare environments, VLC-based MBSN systems address critical challenges such as latency, security, EMI from medical equipment, and health risks associated with exposure to RF technologies. VLC offers significant advantages, including immunity to RF interference, non-interference with medical devices, and enhanced security through eavesdropping prevention. To showcase the practicality of VLC in hospital settings, the authors of [15] implemented a Manchester On-Off-Keying (OOK)-based VLC system in an ICU environment. This system achieved Eye Opening Penalty (EOP) values of 0.89, 0.96, and 2.67 dB over transmission distances of 1.5 m, 5 m, and 15 m, respectively, while successfully monitoring vital parameters such as heart rate, oxygen saturation, and blood pressure, thereby aiding in preventing disease spread. Another study [16] presented a wireless medical assistance system that utilized VLC to overcome RF limitations, improve the transmission of data, and ensure safety in healthcare settings. The design incorporated two MBSNs, an insulin wearable kit, an electrocardiogram (ECG) test device, and emergency remote medical assistance. The study included channel modeling, prototype development, and laboratory testing, all of which demonstrated effectiveness within medical applications. Furthermore, MBSNs collect specific data from wearable sensors placed on patients’ bodies by harnessing WSNs worn on critical parts like the shoulder, wrist, or ankle to obtain optimal vital signs, minimize interference, guarantee comfort, and provide biomechanical stability. Exploiting VLC can help optimize the reliability, efficiency, and security of medical data exchange within healthcare technologies in remote and continuous patient monitoring, personalized healthcare, real-time health data transmission, and implantable medical device development applications. Therefore, providing better diagnostics, treatments, and overall healthcare outcomes represents a major leap toward innovative and patient-centric healthcare solutions [17].

In order to address the practical concerns of implementing MBSN systems in hospital settings, one study [18] offers a comprehensive survey of VLC-based eHealth applications, focusing on modulation and channel coding techniques. While the paper effectively summarizes research efforts that include experimental implementations, it also outlines future challenges such as the need for optimized channel coding and hybrid VLC-RF systems to improve robustness in non-line-of-sight and dynamic hospital conditions. In [19], a detailed review of Internet of Bodies (IoB) technologies explores the modeling difficulties arising from the human body’s complex dielectric properties and identifies electro-quasistatic human body communication as a promising solution, with low signal leakage and high security. It emphasizes the need for advancements in channel estimation, as well as stronger privacy protections. Similarly, ref. [20] reviews IoB communication systems, contrasting RF-based methods with body-coupled communication that offers high data rates and energy efficiency. While both surveys incorporate studies with implementation results, they also underscore ongoing challenges, such as the lack of accurate parametric models, interference in multi-user environments, and the absence of standardized frameworks. These gaps highlight the importance of continued research and regulation to ensure secure, reliable, and interoperable MBSN systems in healthcare settings.

Employing IoT within healthcare brings transformative benefits, such as real-time monitoring and improved health management. However, it also introduces critical ethical challenges. Informed consent is complicated by the passive and continuous nature of data collection in IoT systems, where patients may be unaware of the full scope of data being gathered. Data security is another concern, particularly regarding unauthorized access to sensitive medical information. Data integrity must be maintained to ensure correct diagnosis and treatment, while data privacy risks arise from potential interception during wireless transmission. Furthermore, access control is crucial to prevent misuse by unauthorized parties. To mitigate these risks, encryption protocols, secure cloud computing, and robust access control mechanisms are essential, as shown in [21]. Moreover, the authors of [22] utilized an IoMT interoperable and privacy-focused framework that optimizes network performance and data security within remote healthcare applications. The design includes device authentication, energy-efficient clustering, environmental monitoring, verification of data, and secure encryption. The proposed technique demonstrated a 20% improvement in data rate, a 15% reduction in the rate of packet loss, a 35% increase in network lifetime, and a 10% decrease in both latency and consumption of energy. Additionally, VLC-based MBSNs can enhance further ethical compliance by leveraging their secure communication features to safeguard patient data and ensure ethical compliance in healthcare settings.

In addition, diverse VLC channel parameters such as DC channel gain and Root Mean Square (RMS) delay spread are seriously important in properly enacting overall system performance. Recent advancements in VLC modeling, such as the proposed 3D space–time–frequency geometry-based stochastic model (GBSM), have demonstrated the ability to capture unique indoor VLC channel characteristics, including non-stationarities and the influence of LED radiation patterns and receiver movements, as shown in [23]. The first parameter (DC channel gain) represents transmitted signal attenuation, which has a direct impact on the strength of the received signal and, thus, affects the essential SNR factor. A higher DC channel gain can reduce path loss, but it also leads to more significant signal attenuation over longer distances, affecting the system’s performance by diminishing the received signal power. Additionally, the RMS delay spread characterizes the propagation effect of the multipath within the communication channel, which reflects the received signal temporal dispersion. This temporal dispersion is a direct indicator of multipath effects, where delayed replicas of the transmitted signal interfere with the primary signal, causing Intersymbol Interference (ISI) and degrading communication quality. In VLC-based MBSNs, this parameter provides insight into channel behavior and helps in designing equalization techniques to minimize ISI, enabling higher data rates and reliable transmission in dynamic environments. By understanding and mitigating both parameters, VLC systems can achieve enhanced reliability and efficiency.

In the realm of VLC, multiple developed methodologies are utilized to design robust, efficient communication systems; however, to tackle this challenge, precise estimations of the crucial channel parameters within the VLC environment are a must [24]. That is why VLC presents different valuable approaches, one of which is channel sounding techniques, where the training sequences or the pilot signals are transmitted to characterize the channel response at the receiver.

Moreover, the channel impulse response (CIR) estimation technique can be used to analyze the channel’s response, which is represented as known impulses and transmitted as training sequences or pilot signals. Additionally, different methods, like statistical modeling using Rayleigh or Rician distributions, along with time-domain and frequency-domain analyses, are commonly utilized to estimate crucial parameters such as SNR ratio, delay spread, and multipath propagation. Furthermore, integrating ML to learn intricate mappings and derive channel characteristics in transmitted and received signals has remarkable capabilities for estimating channel parameters in VLC systems. These innovative methods have demonstrated promise in precisely computing channel parameters, therefore enhancing the reliability and efficiency of VLC systems. Utilizing ML-based channel estimation operations offers a data-driven approach that deals with difficult communication environments, which eventually yields more robust and adaptive VLC systems.

### 1.1. ML Approaches for Adaptive Modulation

Based on the aforementioned statements, VLC is a highly promising technology for MBSNs, offering reliable, secure, and high-bandwidth communication. However, challenges persist, particularly signal weakening in dynamic environments. In particular, the body movements of the patient, variations in the distance separating the transmitter and receiver, shadowing, and obstructions can all affect the channel DC gain. Due to these fluctuations, the received signal strength varies, which can introduce errors in the transmitted data [25,26,27].

Adaptive modulation, which dynamically alters the modulation order based on the current channel conditions, is a potential approach to overcome such challenges. This approach enhances spectral efficiency (SE) while ensuring that MBSNs have sufficient communication reliability. With adaptive modulation, modulation schemes can be easily modified to strike an optimal balance between reliability and data rate. While other adaptive modulation methods have been introduced for VLC, this paper focuses on those that use machine learning algorithms. Such approaches that integrate ML use data-based learning and real-time adaptation to dynamic environments, enabling superior system performance optimization. It is important to recognize, however, that ML technique performance might vary over time due to the dynamic characteristics of communication channels.

### 1.2. ML Approaches for Channel Parameter Estimation

Implementing ML algorithms is essential for enhancing the efficiency and robustness of cutting-edge technologies such as VLC systems, addressing real-world challenges, including nonlinear distortion, security vulnerabilities, localization accuracy, jitter, and channel estimation. By leveraging various techniques, ML effectively mitigates fading effects, improves convergence rates, and enhances network resilience against eavesdropping. These algorithms also analyze vast amounts of data to uncover relationships between factors influencing signal propagation, thereby minimizing signal distortion, scattering, and illumination noise. As a result, such models enable systems development with superior location precision, reduced errors, and improved overall performance in VLC deployments [14].

Among these challenges, accurate channel parameter estimation is particularly critical, as it directly influences the system’s ability to model transmission environments, optimize efficiency, and maintain consistent communication under varying conditions. Therefore, in this subsection, we explore several key ML-based approaches that have proven effective in estimating channel parameters for wireless systems. These methods include complex techniques such as k-nearest neighbors (KNN) along with Support Vector Regression (SVR), and the advanced architectures of Recurrent Neural Networks (RNNs), with their variances like vanilla RNN, Gated Recurrent Unit (GRU), and Long Short-Term Memory (LSTM). Each method offers unique advantages, ranging from straightforward interpretability to sophisticated sequential dependencies modeling.

KNN is a supervised non-parametric ML technique used for information estimation and classification. The key concept of KNN is to categorize or forecast results according to how similar the input data points are. This is achieved by comparing data points within the feature space using distance metrics like the Euclidean, Manhattan, Minkowski, and Hamming distances [28]. The output is determined by averaging the values of the k-nearest neighbors for continuous regression tasks, while in discrete classification tasks, the result is found based on the majority class among these neighbors [29].

Moreover, SVR is a supervised ML technique that extends Support Vector Machines (SVMs) to estimate both linear and nonlinear information tasks [30]. SVR minimizes the estimation error by creating a margin called epsilon-tube, which ignores deviations from the true output to help the model focus on the reduction in errors outside of the margin. This approach helps SVR to handle data points more efficiently by concentrating on critical errors rather than optimizing the entire dataset. SVR maps input parameters into higher-dimensional spaces to discover optimal hyperplanes for accurate predictions [31].

Furthermore, RNNs are deep neural network classes frequently utilized in applications that involve sequential data estimation, such as language modeling, text production, speech recognition, time-series forecasting, and video analysis. One of the key features of RNNs is their memory component, which enables them to use previous sequence information to produce new outputs in a sequence [32]. The fundamental form of this architecture is known as vanilla RNN [33], which performs adequately for short sequences where generation depends on the most recent inputs. However, because vanilla RNNs only store data from the most recent few steps, they experience limitations in capturing long-term dependencies when working with longer sequences. This restriction is referred to as the vanishing gradient problem, which prevents the network from effectively propagating information across longer sequences.

In addition, another efficient variant of RNN that has a simplified gate structure is GRU [34]. The gate structure of GRU consists of an update gate ($z_{t}$) and a reset gate ($r_{t}$), which maintain efficiency and performance. Both gates decide the information flow within the ML since they are responsible for how much previous information to use in the next state or ignore from the past output, respectively [35]. Although GRU addresses the vanishing gradient problem and offers moderate computational complexity, it may underperform in certain tasks that involve highly complex long-term sequential dependencies within complicated indoor VLC-based MBSNs healthcare scenarios.

### 1.3. Related Works

Existing research on ML for link adaptation (LA) has primarily focused on communication technologies such as RF [36,37,38,39,40] and underwater acoustic communication systems [41,42,43,44]. While some research has explored learning in VLC, only [45] has specifically examined adaptive modulation in VLC-based MBSNs. However, the author did not consider channel parameter estimation.

Existing ML-driven link adaptation research in RF systems has encompassed various approaches. The deep convolutional neural network of ref. [36] uses per-subcarrier SNR and noise variance as features to predict modulation and coding scheme (MCS) selections without preprocessing, but its very high input dimensionality (hundreds of subcarriers) and reliance on massive offline datasets render it impractical for real-time, resource-constrained body sensors. Ref. [37] applies deep Q-learning, with states defined by a fixed window of recent received signal strength (RSS) measurements and actions as Quadrature Amplitude Modulation (QAM) orders, but it utilizes offline training algorithms, making them impractical for real-time operation. The work in [38] segments the SNR range into static rate regions for deep Q-network (DQN)-based Gray-coded M-ary Phase Shift Keying (MPSK) selection, yet these rigid boundaries and episodic trial strategies risk poor generalization under non-stationary channel statistics. The author of [37] further builds on this by addressing delay propagation in indoor RF environments by introducing a deep Q-learning–based adaptive modulation scheme that incorporates outdated CSI. Moreover, in [39], the author proposes an online deep learning algorithm for massive MIMO that pretrains on Outer Loop Link Adaptation (OLLA) outputs and then incrementally retrains using Acknowledgment and Negative Acknowledgment (ACK/NACK) feedback—leveraging sub-band Signal-to-Interference-plus-Noise Ratio (SINR), Channel Quality Indicator (CQI), Reference Signal Received Power (RSRP), and time-since-sounding as features. However, this approach inherits bias from the initial offline model, assumes full-buffer traffic, and ignores fine-grained feature interactions. Furthermore, the author of [40] introduces a tuning-free Thompson sampling bandit with a latent SINR distribution state and MCS arms, yet their Gaussian-innovation assumption and empirically tuned Doppler smoothing may not hold in line-of-sight–dominated or ambient-light-noisy VLC channels.

Acoustic Underwater Communication (AUWC) systems face substantial challenges due to prolonged propagation delays, which render current CSI obsolete. To mitigate this, ref. [41] proposed a Dyna-Q algorithm for channel state prediction and throughput computation, whereas the authors of [42] designed a Q-learning method incorporating multiple transmission parameters. Additionally, ref. [43] demonstrated that SNR and Bit Error Rate (BER) exhibit weak correlation in underwater channels. In response to the Link Adaptation (LA) issues in AUWC systems, ref. [44] implemented a deep Q-learning technique. Table 2 and Table 3 present previous ML-driven LA research in RF and AUWC systems, respectively [45].

Furthermore, recent research has explored VLC implementations for MBSNs and hospital settings. For instance, ref. [46] investigated patient monitoring systems and MBSNs that utilize VLC and IR data transmission. Meanwhile, ref. [24] focused on assessing VLC system performance for smart patient monitoring. In a different study, ref. [47] examined VLC performance for indoor localization in hospital settings. Furthermore, ref. [18] surveyed recent developments in channel coding and modulation methods, noting that adaptive technologies play a critical role in boosting both reliability and efficiency in dynamic hospital scenarios.

Building on previous work, ref. [45] developed an ML-driven adaptive modulation framework for VLC-enabled MBSNs, specifically targeting the challenges posed by dynamic hospital conditions and patient movement. Their methodology incorporated a sophisticated ray tracing technique to derive CIRs across diverse hospital environments. The author investigated various modulation schemes, including both adaptive and non-adaptive approaches, as benchmarks to improve SE performance. A Q-learning-based modulation approach was chosen for its adaptability to variations in the system and environment, offering dynamic adjustment without requiring explicit CSI. However, the study focused exclusively on modulation techniques and did not address channel parameter estimation.

In order to investigate channel estimation using ML-based VLC systems, ref. [48] explores the usage of an Extreme Learning Machine (ELM) for channel estimation and equalization in VLC systems used in underground mining environments. The proposed ELM-based scheme utilizes single-layer feedforward networks (SLFN) to improve BER performance. Furthermore, the authors of [49] explore the error performance of visible light positioning (VLP) that employs both VLC and indoor positioning systems for 3D indoor drone localization using artificial neural network (ANN)-based ML. The results demonstrate significant accuracy enhancement in drone localization. Similarly, ref. [50] proposes an ML-based VLP system for faster deployment compared to ML-regression techniques within Industrial Internet-of-Things (IIoT) applications by employing an XGBoost-based position estimator. The work in [51] utilizes LSTM to enhance indoor channel estimation within VLC systems. The results demonstrate that the LSTM-based estimator outperforms the traditional Kalman filter (KF) estimator, providing better channel estimation and improved BER. In addition, ref. [52] presents an LSTM-based channel estimation for an optical Intelligent Reflecting Surface (IRS) nonlinear VLC application. The simulation results demonstrated that the LSTM-based method outperforms traditional channel estimation techniques in improving signal detection and reliability, which points out the strong potential for mitigating distortions and maintaining effective communication in realistic VLC environments. Furthermore, the authors of [53] introduce a channel estimation performance comparison of three ML algorithms in a multi-wavelength VLC system. The study showed that the Sparse Autoencoders (SAEs) technique provides the best channel estimation performance compared to other algorithms. Moreover, ref. [54] utilized a hybrid deep neural network (DNN) consisting of multilayer perceptron (MLP), bidirectional LSTM, and GRU for estimation of path loss and jamming detection in a vehicular-based V-VLC environment. The evaluations demonstrated satisfying results in terms of accuracy and error reduction, outperforming current models. Further studies in [55] improved channel estimation by reducing the BER in indoor VLC systems using a comparison between DNN, YOLO v3, and Kalman Filter algorithms, with three different modulation techniques. The results show that DNN performs well over KF, and YOLO v3 optimization enhances channel estimation better than conventional methods. In [56], the authors introduce new Random Fourier Features (RFFs)-based ML within a nonlinear VLC channel. The results show that RFF-based ML performs with lower training approximation and better classification accuracy, particularly in data-scarce environments. In addition, ref. [57] overviews the utilization of Federated Learning (FL) within VLC systems to address challenges like privacy concerns and communication performance in traditional centralized ML approaches, outlining key design aspects aimed at improving system robustness and efficiency. Table 4 presents a summary of the existing ML-based VLC channel estimation techniques.

### 1.4. Contributions

Building upon this groundwork, the key contributions of this paper are summarized as follows:

- We built upon the ray tracing technique proposed in [58] to derive CIRs in real hospital layouts, seamlessly incorporating user-random mobility parameters, artificial structures, wavelength-dependent diffuse and specular reflections, actual light sources, and up to 10 reflection orders, all while satisfying illumination standards. This approach allows for more accurate modeling of complex indoor VLC propagation conditions in healthcare environments.
- We developed a Q-learning scheme for DC-biased optical Orthogonal Frequency Division Multiplexing (DCO-OFDM), with intensity modulation and direct detection (IM/DD), addressing the challenge of meeting varying QoS demands in 6G VLC-enabled healthcare monitoring systems.
- We designed ML-based algorithms to estimate PL and RMS delay spread in VLC-based MBSNs, improving reliability and supporting robust 6G health monitoring applications.

The rest of the paper is organized as follows: Section 2 provides the system model, Q-learning-based adaptive modulation framework, and LSTM-driven channel-parameter estimation method. Section 3 details the key simulation results. Section 4 concludes our findings.

## 2. System Model

### 2.1. Mobile Channel Model for VLC-Based MBSNs

In order to accurately model VLC channel characteristics, various methods are utilized, with Zemax^®^ (SMART Research Program L113955) ray tracing software being a prominent approach [58]. Within the software, the sequential ray tracing method traces rays between the transmitter and receiver through a sequence of surfaces, with each surface being hit only once, making it ideal for imaging systems. On the other hand, the non-sequential ray tracing technique allows rays to reflect and scatter multiple times in any order throughout the environment. This flexibility enables the modeling of more realistic propagation scenarios that account for complex interactions with human bodies, furniture, and medical equipment. By accurately capturing these interactions, the non-sequential approach provides a more comprehensive estimation of the CIR, leading to higher accuracy and reliability [59].

**Figure 3:**
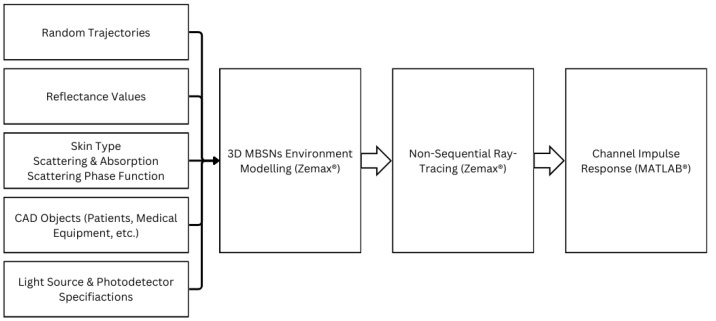
Site-specific channel modeling steps for VLC-based MBSNs.

**Figure 4:**
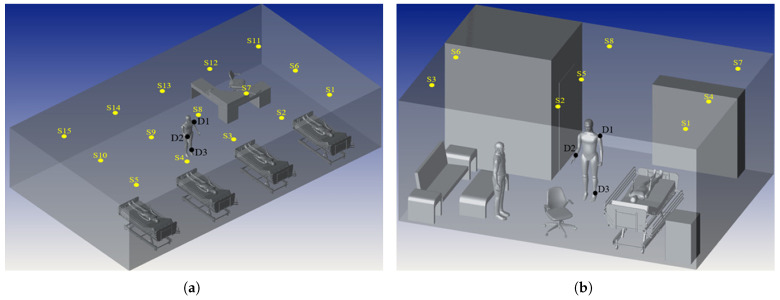
Hospital settings: (**a**) ICU ward and (**b**) FTPR [61].

Therefore, this paper adopts the site-specific non-sequential ray tracing method described in [60] and summarized within Figure 3. The 3D hospital scenarios demonstrated in Figure 4 were initially constructed using real-life data by arranging CAD objects to reflect realistic hospital environments. Additionally, the reflectances of CAD object surfaces were specified to account for wavelength dependence. The layout of luminaires and photodetectors (PDs) was then organized with specifications tailored to VLC applications. The orientation parameters of sensor nodes coupled with detectors on the shoulder, wrist, and ankle, respectively, were adjusted based on the body position at each sample point along a trajectory.

Previous channel modeling studies often rely on classical mobility models, such as random user locations. While these simplified assumptions may be suitable for infrared (IR)-based channels, they are insufficient for accurately modeling VLC systems. In VLC-based MBSNs, capturing realistic human mobility is essential, as individuals do not walk with constant stride lengths, follow only cardinal directions, or start new paths strictly from doorways. To address these limitations, we adopt a more realistic random trajectory model that better reflects natural human movement. This, combined with wavelength-dependent channel modeling, enables practical characterization of dynamic VLC channels in healthcare environments [60]. Accordingly, a random trajectory generator is utilized to produce the realistic mobility patterns of a user within the considered scenarios. While the model focuses on two specific hospital settings, it can be designed to accommodate various other hospital settings as well. The trajectories are represented as multiple sample points across different paths, considering random step lengths, directions, and starting points. This ensures the output model’s performance is reliable across different assumptions, including varying user mobility and hospital settings.

In order to mitigate the photodetector saturation effects caused by exposure to various ambient light sources, such as artificial lighting and sunlight, robust techniques have been explored. In [62], the authors used direct current optical orthogonal frequency division multiplexing with adaptive bit and energy loading, along with optical bandpass blue filters for VLC systems under solar irradiance. The results showed data rates exceeding 1 Gb/s under solar illuminance of 50,350 lux without optical filtering. Furthermore, using off-the-shelf blue filters enhanced the SNR ratio by at least 6.47 dB, compensating for approximately 50% of the reduced data rate. This technique could be adapted within our model to address potential photodetector saturation effects and ensure reliable performance under high ambient light conditions.

Ray tracing simulations provide data on total travel distance and received power for each launched photon from a source to a PD. These simulations are processed using MATLAB^®^ (R2024b) to compute CIRs as

(1) $$h\left(t\right)=\sum_{k=1}^{M}P_{k}\;\delta \left(t-t_{k}\right)$$

where $P_{k}$ represents the detected power of the $k^{th}$ ray, $t_{k}$ denotes its travel duration, and *M* is the total number of collected rays.

**Figure 5:**
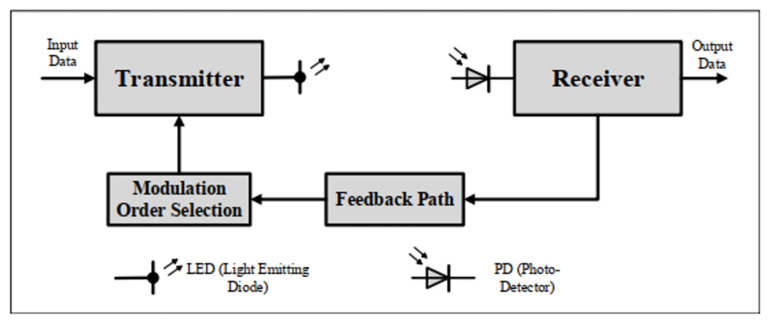
VLC-based MBSNs system model [45].

In MBSN systems, due to strict power and size limitations, on-body sensor nodes must be designed with minimal complexity. Therefore, the selection of the modulation order is handled on the transmitter side in the proposed system model, as per Figure 5. This VLC system uses M-ary Pulse Amplitude Modulation (PAM) with a realistic CIR, expressed as [27]

(2) $$s\left(t\right)=2P_{avg}\sum_{i}m_{i}p\left(t-iT\right)$$

where s(t) is the modulated signal, $P_{avg}$ indicates the average optical power, $m_{i}\in \left\{m/\left(M-1\right)|m=0,1,\ldots ,M-1\right\}$ is the amplitude of the $i_{th}$ symbol, p(t) is the pulse shape with $T^{-1}\int p\left(t\right)dt=1$ and p(t)=0 for t∉[0,T], and *T* is the symbol duration. Transmitted light is modulated by s(t) and then passes through the channel. The received signal of the PD can be mathematically represented by the following expression:

(3) r(t)=s(t)∗h(t)+n(t).

The noise component, n(t), accounts for background interference and shot noise, both assumed to be white and Gaussian in nature. Consequently, ISI is eliminated at the receiver end. The photocurrent received at the PD output is expressed as follows:

(4) $$\begin{array}{cc} I(t) & =\sum_{k=1}^{K}P_{k}s\left(t-t_{k}\right)+n\left(t\right)\\ \; & =\sum_{i}2RP_{avg}m_{i}\sum_{k=1}^{K}P_{k}p\left(t-iT-\tau_{k}\right)+n\left(t\right). \end{array}$$

Here, *R* represents the responsivity of the PDs. Since no explicit mathematical expression exists for the indoor VLC channel model, simulation under specific conditions is required. The simulation in [60] was performed by utilizing a site-specific non-sequential ray tracing approach across two different hospital settings. Figure 4 demonstrates the placement of three photodetectors on the mobile patient’s ankle, shoulder, and wrist across FTPR and ICU ward environments. The patient moves along random trajectories, and the received CIR is simulated for every PD. Maximizing throughput while maintaining the SER within a specified constraint along these paths is the primary objective. This is carried out by strategically modifying the order of PAM. Thus, the optimization problem for adaptive modulation can be defined as follows:

(5) $$arg max\mu \in I\left\{R_{\mu }:SER_{\mu }\leq SER_{tar}\right\}$$

where $R_{\mu }$ represents the throughput achieved with a specific modulation order. The set *I* encompasses all possible modulation orders, represented by μ. $SER_{\mu }$ represents the instantaneous SER for μ, whereas $SER_{tar}$ denotes the maximum acceptable SER.

The main problem in Equation (5) is that the modulation order μ can only take on discrete values. This makes the optimization inherently combinatorial. Specifically, the discrete modulation order μ∈I={2,4,…,64} transforms the optimization problem in Equation (5) into a combinatorial search over a finite action set. Although the set is limited in size, exhaustive search becomes impractical for on-body sensor nodes operating under stringent computational and energy constraints. Furthermore, the $SER_{\mu }$ is a highly nonlinear function of the instantaneous channel impulse response h(t) and received signal-to-noise ratio ρ, both of which vary dynamically due to patient movement, body shadowing, and multipath effects in indoor medical environments. As there are no closed-form expressions for BER or throughput under the ray-traced VLC channel model used in [60], the feasible region $\left\{\mu :SER_{\mu }\leq SER_{tar}\right\}$ is inherently non-convex and must be evaluated numerically. These characteristics eliminate the applicability of convex or gradient-based optimization methods and necessitate a model-free, lightweight decision-making approach that supports real-time adaptation while satisfying power and latency limitations.

Q-learning does not require any explicit channel model or CSI estimation, making it ideal for VLC-MBSNs, where the CIR varies unpredictably with patient movement and ambient light changes. Moreover, the discrete set of PAM orders maps naturally to the action space of Q-learning. Classical convex or gradient-based methods cannot handle such integer choices effectively, whereas Q-learning’s table-based update over (state, action) pairs directly accommodates discrete modulation levels. Furthermore, on-body sensors are energy- and compute-limited. Q-learning’s core operations are simple lookups and updates, unlike deep-learning models that demand significant memory and cycles. This makes real-time, on-device adaptation feasible without offloading. Q-learning learns by trial and error during operation, continually refining its policy. It naturally accommodates non-stationary channels, whereas supervised methods generally lack mechanisms to adapt post-deployment.

The VLC channel for MBSNs is characterized by its DC gain, given by

(6) $$H_{0}=\int_{0}^{+\infty }h\left(t\right)dt.$$

Then, the path loss is calculated using

(7) $$PL=-10\log_{10}H_{0}.$$

The RMS delay spread, representing the standard deviation of the delays, is another key channel characteristic, defined as

(8) $$\tau_{RMS}=\;\sqrt{\frac{\int_{0}^{+\infty }{\left(t-\tau_{0}\right)}^{2}\;h\left(t\right)dt}{H_{0}}}\;$$

where $\tau_{0}$ denotes the mean excess delay

(9) $$\tau_{0}=\frac{\int_{0}^{+\infty }t\cdot h\left(t\right)dt}{H_{0}}.$$

The statistical models proposed in [61] for PL and RMS delay spread for realistic ICU ward and FTPR settings. Specifically, 20 random trajectories were analyzed, each consisting of 10 consecutive points per scenario. Successive steps in a trajectory were created using randomly chosen starting points, directions, and step lengths. Furthermore, the mobile user advances toward the next sample point at each position along the trajectory [60]. The width *A* and length *B* of the considered scenario and the matrix C_2×2_, which stores the boundaries of the valid area in a hospital room, are initialized. It is crucial to note that the step direction angle ϕ is chosen uniformly. The matrix D_10×2_ stores the coordinates of each sample point randomly generated along the trajectory. The algorithm then verifies whether the points on the trajectory lie within the eligible region. Then, the CIR at each sample point is determined for every photodetector on the mobile user. The extracted CIRs are used to compute the PL and RMS delay spread. The author of [61] then visualized the obtained PL and RMS delay spread through histograms accompanied by best-fit curves for D1–D3 in both the ICU ward and FTPR. The random trajectory generator algorithm is described in Algorithm 1.

Extensive simulation studies, as described in [61], demonstrate that the log-normal distribution provides a good fit for both path loss and RMS delay spread histograms, as given by

(10) $$f\left(PL\right)=\frac{1}{PL\;\sigma \sqrt{2\pi }}\;\;\exp\left(-\frac{{\left(\ln\left(PL\;\right)-\mu \right)}^{2}}{2\sigma^{2}}\right)$$

(11) $$f\left(\tau_{RMS}\right)\;=\;\frac{1}{\tau_{RMS}\;\sigma \sqrt{2\pi }}\;\;\;\exp\;\left(\;\;-\frac{{\left(\ln\left(\tau_{RMS}\;\right)-\mu \right)}^{2}}{2\sigma^{2}}\right)\;$$

where μ and σ denote location and scale parameters, respectively.
**Algorithm 1:** Random Trajectory Generator
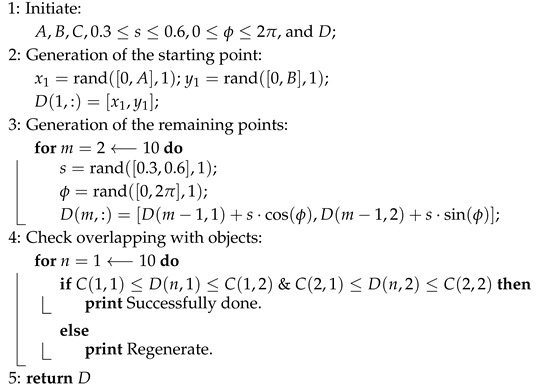


### 2.2. Proposed Q-Learning-Based Adaptive Modulation Scheme

Adaptive modulation presents a complex challenge within the context of RL due to the volatile and dynamic characteristics of the VLC-driven MBSN system. We start by providing a brief overview of RL and then delve into the Q-learning-based adaptive modulation scheme.

#### 2.2.1. Reinforcement Learning-Based Adaptive Modulation

Reinforcement learning is an ML approach focused on an agent’s dynamic engagement with its surroundings, aiming to develop optimal decision-making strategies that accumulate maximum rewards over time. Unlike supervised learning’s reliance on comprehensive labeled datasets, RL agents acquire knowledge through continuous trial and error.

Among popular RL algorithms, Q-learning is frequently employed to handle Markov Decision Processes (MDPs). Grasping Q-learning starts with understanding its foundational components. *S* represents the state space, which includes the perceived states *s* that the agent observes in the environment. Moreover, *A* defines the action space, specifying the set of possible actions *a* that the agent is able to perform in every state. Then, the immediate reward function, r(s,a), determines the reward acquired once the agent performs a specific action in a given state. Furthermore, π(s) represents the policy, which defines the mapping between observed states and the corresponding actions for the agent. According to the selected policy, the Q-function Q(s,a) estimates the cumulative future reward, discounted over time, that results from taking a particular action in a given state. The algorithm then updates the Q-values through the following process:

(12) $$Q\left(s,a\right)⟵Q\left(s,a\right)+\alpha \left[r\left(s,a\right)+\gamma \underset{a^{\prime }\in A,s^{\prime }\in S}{arg max}Q\left(s^{\prime },a^{\prime }\right)-Q\left(s,a\right)\right]$$

where α∈[0,1] represents the learning rate, γ∈[0,1] denotes the discount factor, $s^{\prime }$ denotes the next state, and $a^{\prime }$ represents the possible actions. At its core, Q-learning strives to derive an optimal policy such that, over time, the expected cumulative reward is maximized. This optimal policy uses the following expression:

(13) $$\pi^{*}\left(s\right)=\underset{a\in A}{arg max}Q(s,a).$$

One widely used method for balancing exploration and exploitation is the ϵ-greedy strategy.

#### 2.2.2. Q-Learning-Based Adaptive Modulation

**Figure 6:**
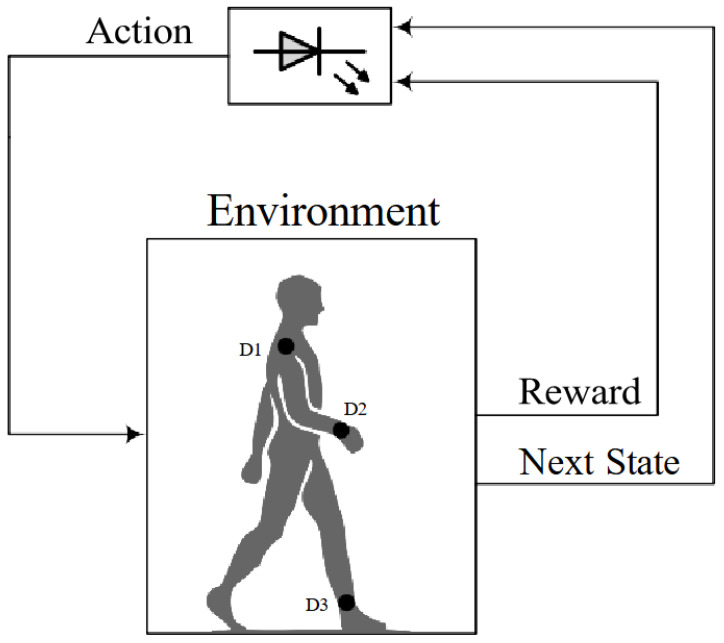
Reinforcement learning model applied to adaptive modulation in VLC-based MBSNs [45].

For the adaptive modulation optimization problem, the tuple ($H_{0}$, ρ) is defined as the state space, where the action space comprises the available modulation orders, and ρ denotes the quantized received signal-to-noise ratio. Consequently, when the agent modifies the modulation order for a specific channel state, it encounters a new state within the state space. By formulating the problem as an MDP, it becomes suitable for a solution using the Q-learning algorithm. Figure 6 demonstrates how patient mobility and agent actions jointly drive state transitions.

Our model does not account for state changes resulting from human movements. Instead, the MDP for Q-learning-based adaptive modulation involves state transitions driven solely by the decisions made by the agent under the current CIR. It is important to emphasize that the speed of the patient is slow enough to allow the agent to explore each state thoroughly. Moreover, after training is completed, the agent chooses the modulation order based on initial channel observations. The received SNR is given by

(14) $$\rho =\frac{P}{\sigma_{n}^{2}}{\left|H_{0}\right|}^{2}.$$

Here, *P* denotes the transmitted optical power, $\sigma_{n}^{2}$ represents the noise power, and $H_{0}$ refers to the channel DC-gain, which can be expressed as

(15) $$H_{0}=\int_{0}^{+\infty }h\left(t\right)dt=\sum_{k=1}^{M}P_{k}$$

where *M* and $P_{k}$ are defined in Equation (1).

r(s,a) represents the reward function, which measures the throughput resulting from taking action *a* in state *s* within the given environment; it is given as follows:

(16) $$r\left(s,a\right)=\left\{\begin{array}{cc} \log_{2}\left(\mu \right)\left(1-SER_{\mu }\right), & \mathrm{if}\;SER_{\mu }\leq SER_{tar}\\ -SER_{\mu }, & \mathrm{if}\;SER_{\mu }>SER_{tar} \end{array}\right.$$

Here, $SER_{tar}$ represents the required target symbol error rate. Furthermore, the ϵ-greedy approach is used, with a high initial ϵ value to facilitate exploration in the early learning stages. During the early stages of learning, the agent selects random actions, gaining valuable insights into the environment. Over time, ϵ is decreased to favor exploitation over exploration, encouraging the agent to follow the learned policy. Algorithm 2 outlines the introduced Q-learning-based adaptive modulation scheme. The proposed algorithm leverages a lightweight, model-free Q-learning agent to dynamically select the optimal PAM order based solely on quantized channel gain and received SNR feedback, eliminating the need for explicit CSI or complex channel models. Its strength lies in balancing exploration and exploitation through an ϵ-greedy policy, which enables real-time adaptation on resource-constrained on-body sensors while consistently meeting the target SER and maximizing throughput.
**Algorithm 2:** Q-learning-based Adaptive Modulation for VLC-based MBSN
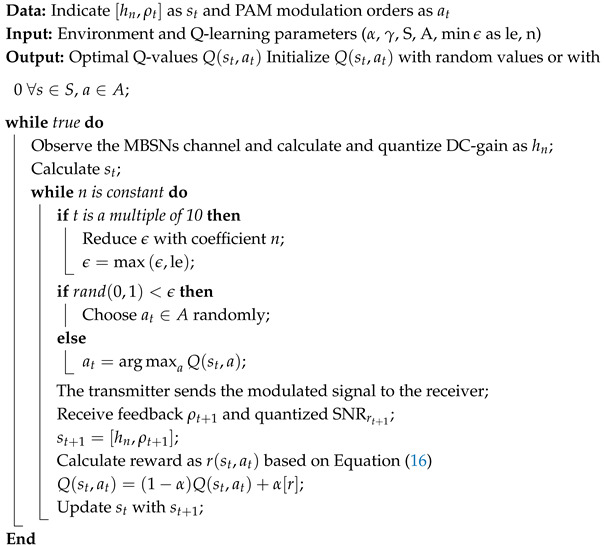


### 2.3. Proposed LSTM-Based Channel Parameter Estimation

**Figure 7:**
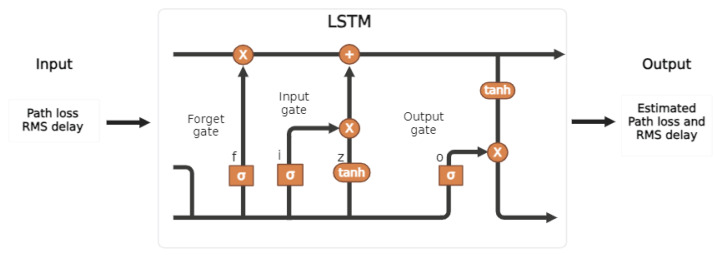
LSTM architecture to estimate the PL and $\tau_{RMS}$ of VLC-based MBSNs.

Long Short-Term Memory (LSTM) is a special RNN type consisting of an input gate $i^{\left(t\right)}$, forget gate $f^{\left(t\right)}$, cell gate $c^{\left(t\right)}$, and output gate $o^{\left(t\right)}$[63]. This algorithm allows for the prediction of random walks by the user over random trajectories without knowing the sample points, and it also resolves the vanishing gradient problem. The general structure is illustrated in Figure 7. Furthermore, LSTM is capable of handling complex and dynamic propagation environments with higher prediction accuracy, adaptability, and performance, unlike traditional methods [64]. LSTM networks excel in handling sequential data, where the order of data points is both significant and highly correlated. They are designed to iteratively learn these correlations, enabling them to estimate future data points based on past observations. This capability, combined with their memory cell and gating mechanism, allows LSTM to effectively capture long-range temporal dependencies and adapt to the continuous fluctuations of wireless communication channels. Even in scenarios with high variability over time, their ability to selectively remember or forget information makes them an ideal choice for modeling dynamic channel conditions and user mobility in VLC-based MBSNs. LSTM starts by updating the block input using the current information $x^{\left(t\right)}$ together with the last LSTM output $y^{\left(t-1\right)}$ in the form of

(17) $$z^{\left(t\right)}=g\left(W_{z}x^{\left(t\right)}+R_{z}y^{\left(t-1\right)}+b_{z}\right)$$

where $W_{z}$, $R_{z}$, and $b_{z}$ are the weights for the input, output, and the bias weight vector, respectively. The estimated information could be found by the current cell value and the output gate, as follows:

(18) $$y^{\left(t\right)}=g\left(c^{\left(t\right)}\right)⊙o^{\left(t\right)}$$

where ⊙ is the point-wise multiplication of two vectors along with g(x)=tanh(x). The algorithmic details are outlined in Algorithm 3, which calculates the gradients necessary for adjusting the weights within each gate.

In evaluating the performance of the ML-based system, we utilized Root Mean Squared Error (RMSE) as the loss function. RMSE is widely favored in VLC-based MBSNs for its ability to directly quantify the accuracy of channel parameter estimations, such as path loss and RMS delay spread. By highlighting errors in the estimated values, RMSE provides crucial insights into how well the system models real-world conditions. The RMSE function is mathematically defined as follows:

(19) $$RMSE=\sqrt{\frac{1}{n}\sum_{j=1}^{n}{\left(y_{j}-\hat{y_{j}}\right)}^{2}}$$

where $y_{j}$, $\hat{y_{j}}$, and n represent the actual data, estimated data, and number of data points, respectively.
**Algorithm 3:** ML LSTM-based Path Loss and RMS Delay Spread Estimation for VLC-based MBSNs
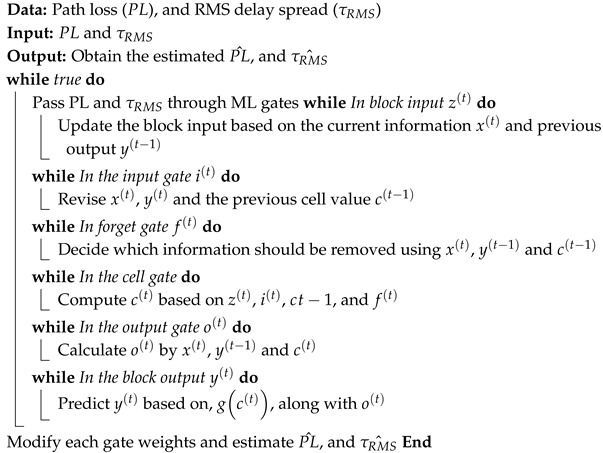


Furthermore, the path loss and RMS delay spread were used as input features for training the LSTM. The dataset was split into an 80% training set and a 20% validation set. Data preprocessing, including normalization, was applied to improve training stability and enhance the model’s performance. Moreover, the LSTM architecture consists of 55 neurons in the hidden layer, designed to balance model complexity and the capacity to represent patterns in the sequential data. A dropout layer with a rate of 0.4 was introduced to mitigate overfitting, followed by a fully connected layer and a regression layer to estimate the real value output. The model was trained using the Adam optimization algorithm, which effectively controls training speed, convergence, and generalization performance. The training was conducted over 400 epochs to ensure robust learning. These parameters were selected based on the practical implementation of ML in VLC systems, ensuring their relevance and applicability to real-world scenarios, such as [65]. Other design characteristics of the LSTM are presented in Table 5.

To determine the time complexity of the designed LSTM model, let *B* represent the effective batch size during training, *H* the number of hidden units, and *F* the number of input features. The total number of operations performed per iteration is approximately given by O(BH(F+H)).

## 3. Simulation Results

A site-specific non-sequential ray tracing technique [58] is employed within the ICU ward and FTPR hospital scenarios to find the CIRs. Both scenarios utilized CAD objects to obtain the dependent wavelength reflectances and the specific luminaries on the ceilings and PDs arranged within the human body. The luminaries selected are distributed to ensure the minimum uniformity illuminance ratio and minimum average illumination level. Moreover, three node sensors are attached to the mobile human where (D1) is positioned on the shoulder, (D2) on the wrist, and (D3) on the ankle to form the MBSNs [58]. The first room is an ICU ward with four patients in their beds, a healthcare provider who walks randomly within the room, a chair, and a desk. Furthermore, the second scenario is an FTPR with a patient in the bed, a healthcare provider who is also considered walking randomly in the room, furniture, a sofa, and a restroom. The ICU ward has 11.5 m × 6.5 m × 3 m room dimensions with 15 luminaries on the ceiling, whereas the FTPR has 7 m × 5 m × 3 m dimensions with 8 luminaries. Furthermore, 20 random trajectories with 10 successive points in each scenario are considered, and the step length and direction are uniformly selected. After generating the random trajectory movements, path loss and RMS delay spread are obtained from the CIR, which considers real-life specifications and serves as inputs for different ML algorithms to estimate PL and RMS delay spread.

### 3.1. Q-Learning-Based Adaptive Modulation

In this study, the CIRs obtained from a previous work [60] were used. The evaluation focused on SE performance across multiple schemes: the Q-learning-based adaptive modulation, the KNN-based adaptive modulation, a non-adaptive scheme, and the optimal achievable SE. Additionally, for all channels, a flat fading channel model is used, given its relevance for the low data rates characteristic of MBSN applications, which has demonstrated satisfactory results for the study. The parameters for the adaptive modulation algorithm and a summary of the system model are presented in Table 6.

**Figure 8:**
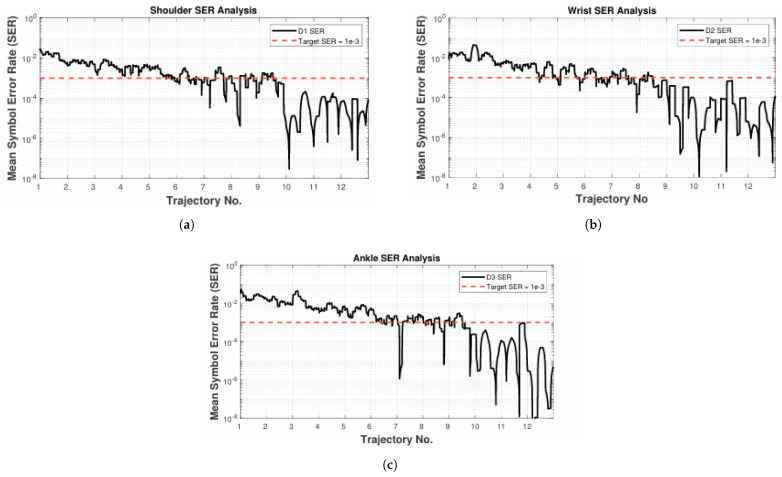
Training stage of Q-learning-based adaptive modulation scheme in the ICU ward. (**a**–**c**) correspond to D1–D3, respectively.

The Q-learning-based modulation scheme does not require CSI for its model training; instead, it acquires knowledge by extensively exploring its environment. Even as the exploration factor gradually diminishes, exploration continues, allowing dynamic adjustment to changes in both the system model and its environment. The algorithm fundamentally relies on these two properties. As depicted in Figure 8, during the initial stages of training, the Q-learning-based adaptive modulation starts with an exploration phase, which results in an initial SER that is higher than the intended target. Over time, the SER steadily declines. After accumulating sufficient information in the Q-table, the agent shifts to making more deterministic choices through the use of a greedy strategy. Furthermore, when the system adopts greedy decision-making, the SER does not experience a significant drop; instead, it fluctuates just below the $SER_{tar}$. Since excessively low SER values are not considered ideal, this outcome aligns with the goal of optimizing SE.

**Figure 9:**
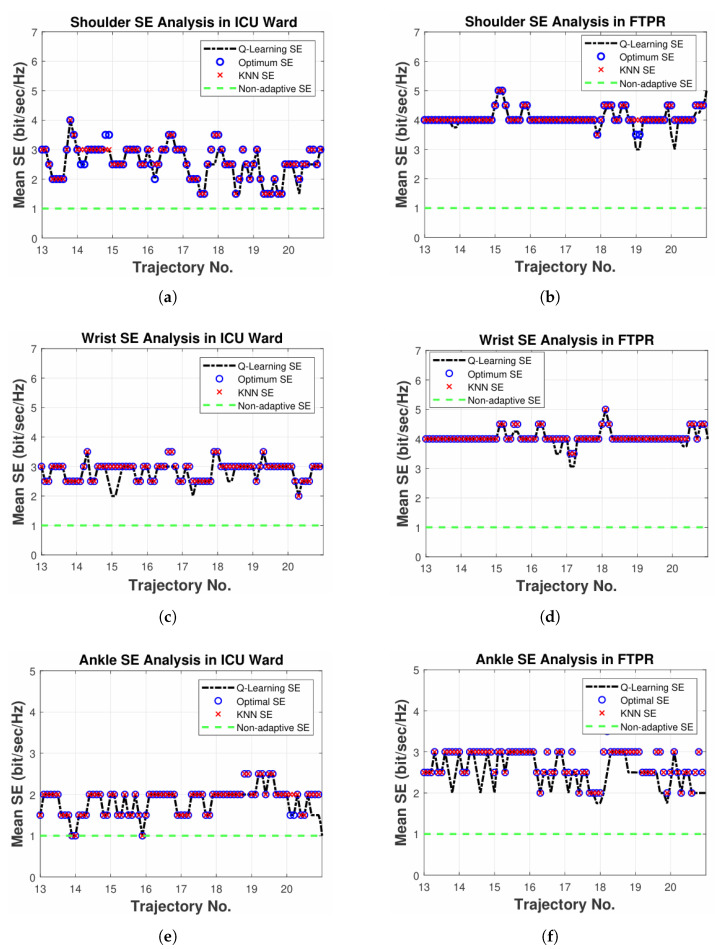
Spectral efficiency analysis of various modulation schemes in (**a**,**c**,**e**) ICU ward and (**b**,**d**,**f**) FTPR [45].

The SE performance of various methods is depicted in Figure 9. Optimal SE—defined as the maximum SE that still fulfills the necessary $SER_{tar}$—is the benchmark for performance. In this scenario, the KNN approach is configured to utilize 60% of the CIRs, corresponding to 12 trajectories, with K set to 3 for nearest neighbor calculation. Unlike the non-adaptive method that resorts to binary PAM to achieve the target $SER_{tar}$, both the KNN and Q-learning strategies bring about considerable improvements in SE. As illustrated in Figure 9a,b,e, there are instances where the KNN method’s SE surpasses the optimal level, suggesting that the desired $SER_{tar}$ is not achieved in those occurrences.

Unlike other methods, the Q-learning approach consistently satisfies the desired $SER_{tar}$ in all figures. Nonetheless, the SE may fall short of the optimal value in some instances as a result of quantization level limitations, particularly when the optimal SER is near $SER_{tar}$. In these cases, the method favors meeting the $SER_{tar}$ target, taking a more conservative approach. Although raising the quantization levels improves precision, it comes at the cost of greater complexity. Additionally, the continuous exploration process contributes to this behavior.

In addition, Significant SE improvements are observed when employing a Q-learning-based adaptive modulation scheme over a non-adaptive approach. In the ICU ward, the observed increases are 151%, 178%, and 81% for D1, D2, and D3, respectively. Additionally, our model exhibits substantial SE gains within the FTPR scenario, specifically achieving 304%, 303%, and 151% for D1, D2, and D3, respectively. This higher SE improvement in the FTPR scenario, in contrast to the ICU ward, indicates that the channel DC gain range in FTPR is significantly broader, which is consistent with the results reported in [60].

Moreover, PDs placed on the shoulder (D1) and wrist (D2) show greater SE improvements with the learning-based adaptive modulation approach, in contrast to those placed on the ankle (D3), across both scenarios. The disparity results from the sinusoidal pattern of the DC gain in D1 and D2, produced by their line-of-sight (LOS) rays. Unlike D1 and D2, D3 is mostly influenced by NLOS rays, producing a smoother DC gain pattern. Due to the narrow range of DC gain, D3 exhibits decreased SE compared to other nodes.

In high-dynamic healthcare settings such as emergency wards, Q-learning adaptive modulation faces certain challenges. Since Q-learning relies on sufficient exploration to learn optimal actions, if the channel state changes faster than the agent can explore, the Q-table may never converge to a good policy. To solve such limitations, immediately revert to the most robust, lowest-order modulation (e.g., 2-PAM). Though throughput is reduced, this guarantees SER targets without relying on incomplete training.

Several studies have proposed robust fallback strategies to address rapidly changing environments. The author of [66] introduced a pseudo-reward-based fallback policy approach, in which multiple “pseudo-agents” are trained concurrently alongside the primary policy by augmenting the standard reward with a distance-based pseudo-reward term; at run-time, the system can switch to whichever fallback policy best matches the current state-space distribution, which results in a conservative operation when the optimal policy is unreliable. In the context of resource-constrained devices, ref. [67] proposes an optimized exploration guidance mechanism that aggressively penalizes Q-values associated with repeatedly failing actions, coupled with bootstrapped Q-table initialization, where initial Q-values are set to heuristic estimates proportional to inverse distance to performance targets, and an adaptive ϵ-greedy schedule escalates exploration after errors and decays it during stable conditions. Together, these techniques accelerate convergence, bias initial action selection toward safer modulation orders, and dynamically balance exploration and exploitation under rapidly changing channel conditions.

### 3.2. LSTM-Based Path Loss and RMS Delay Spread Estimation

**Figure 10:**
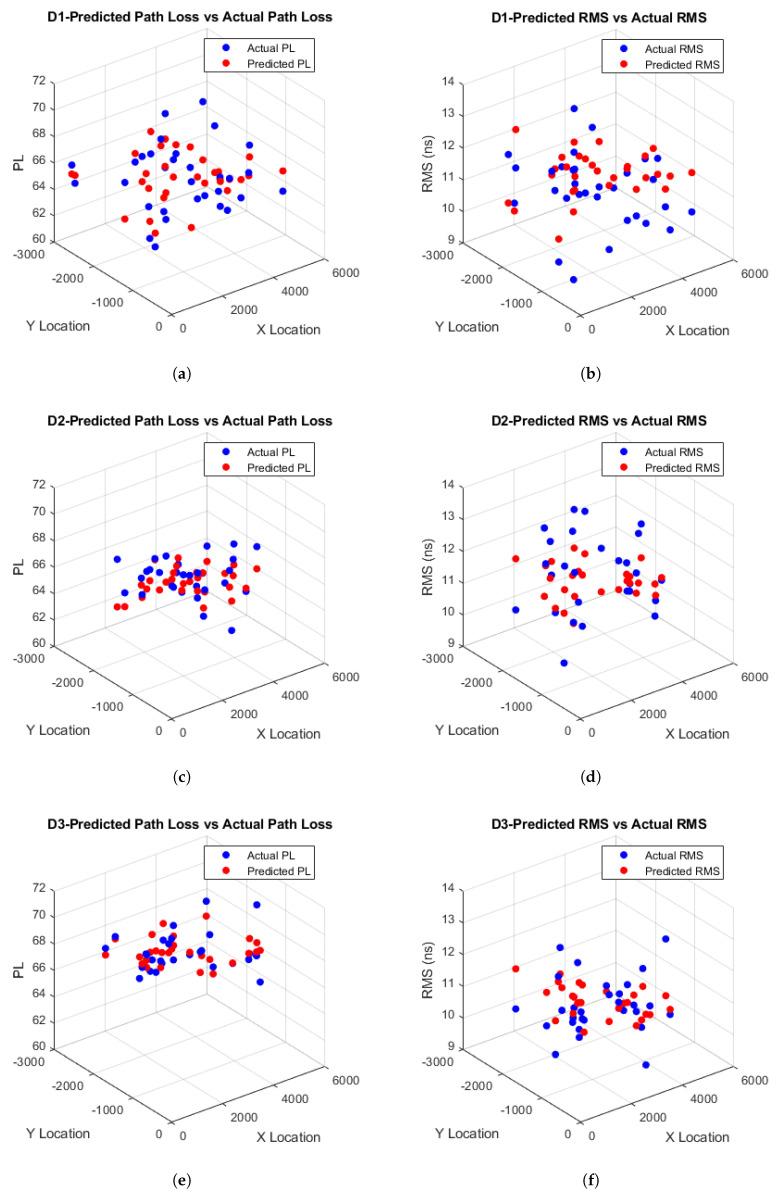
(**a**,**c**,**e**) Estimated path loss and (**b**,**d**,**f**) RMS delay distribution in the ICU ward.

**Figure 11:**
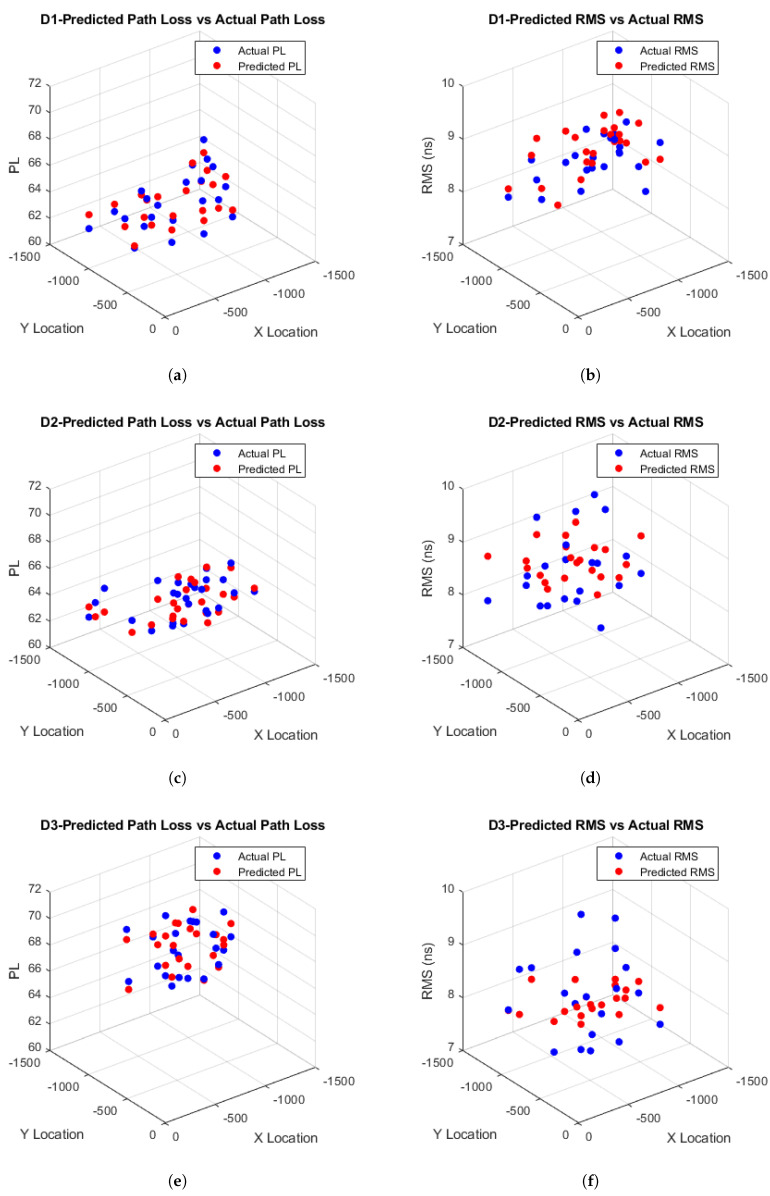
(**a**,**c**,**e**) Estimated path loss and (**b**,**d**,**f**) RMS delay distribution in FTPR.

After comprehensive simulation results using various ML techniques, the estimated path loss and RMS delay spread for D1–D3 in both ICU ward and FTPR scenarios were obtained. The observed RMSE values for these scenarios are detailed in Table 7 and Table 8. The LSTM algorithm consistently outperforms other models in both hospital settings, achieving the lowest RMSE for path loss and RMS delay spread, as illustrated in Figure 10 and Figure 11. This demonstrates the superior performance of LSTM in minimizing prediction errors.

Based on Table 7, it is observed that the LSTM model consistently yields the lowest RMSE values for both PL and RMS spread across all detectors (D1–D3) within the ICU ward, outperforming GRU, vanilla RNN, SVR, and KNN, which exhibit progressively higher errors. Specifically, for PL in the ICU setting, LSTM achieves RMSE values of 1.6797, 1.1679, and 1.1464 at D1, D2, and D3, respectively, compared to the closest ML, which is GRU with values of 1.7060, 1.1808, and 1.1774. Furthermore, based on Table 8, LSTM, again, achieves the smallest RMSE for PL in the FTPR scenario, with respective values of 0.7210, 0.7327, and 1.0652 at D1, D2, and D3, whereas GRU reports slightly higher RMSEs of 0.7359, 0.7832, and 1.1480. In addition, based on Table 7, LSTM RMS delay spread in the ICU ward yields RMSEs of 1.0567, 0.9348, and 0.8784 at D1, D2, and D3, compared to the closest values of GRU of 1.0794, 0.9593, and 0.8840. Similarly, based on Table 8 for the FTPR setting, LSTM records the lowest RMSE values of 0.5830, 0.6230, and 0.7657 at D1, D2, and D3, outperforming the nearest results of GRU of 0.6183, 0.6352, and 0.8555. The remaining techniques follow on in increasing order of RMSE for both settings and in both PL and RMS spread.

Furthermore, based on Table 7, it is observed that the estimated path loss for D1 within the ICU ward scenario has the highest RMSE compared to D2 and D3, confirming the results in [61], where the log-normal distribution of D1 has the highest variance value of 0.0262 compared to D2 and D3, with variances of 0.0176 and 0.0169, respectively, since higher variance results in higher estimated RMSE. Furthermore, based on Table 8, it is observed that D3 has the highest RMSE compared to D1 and D2 within the FTPR scenario, which also confirms the results in [61], where the log-normal distribution of D3 has the highest variance value of 0.0168 compared to D1 and D2, with variances of 0.0123 and 0.0119, respectively, since the highest detector variance shows higher estimated RMSE.

From Table 7, it is also observed that the estimated RMS delay spread for D1 within the ICU ward scenario has the highest RMSE compared to D2 and D3, which is expected, as the log-normal distribution of D1 obtained in [61] has the highest variance of 0.0975, while D2 and D3 have variances of 0.0847 and 0.0780, respectively, indicating that the higher variance of D1 contributes to its increased estimated RMSE. However, based on Table 8, it is observed that D3 has the highest RMSE compared to D1 and D2, confirming the results in [61], where the log-normal distribution of D3 has the highest variance value of 0.0967 compared to D1 and D2, with variances of 0.0659 and 0.0747, respectively, further illustrating that the higher variance leads to a higher estimated RMSE.

A practical complexity analysis is presented to verify the selection of LSTM, evaluating both training and prediction times within the ICU ward and FTPR settings, as shown in Table 9 and Table 10, respectively. The focus is directed toward LSTM, GRU, and RNN, given their advantages and extensive use in MBSN applications. These models, as we stated before, are designed for sequential data regression tasks, excelling at capturing temporal dependencies; this makes them well-suited for time-series prediction and real-time health monitoring.

Based on Table 9, it is found that within the ICU ward, LSTM outperforms GRU and RNN in terms of execution time for D1–D3 across both PL and RMS delay spread, thereby verifying the choice of LSTM. The execution times for D1–D3 PL within the ICU ward were 68.051 s, 65.854 s, and 66.229 s, respectively, while the RMS delay spread execution times for the ICU ward were 69.946 s, 68.786 s, and 68.948 s, respectively. Similarly, based on Table 10, the analysis results indicate that LSTM is also preferable within the FTPR for D1–D3 in terms of execution time for both PL and RMS delay spread. The execution times for D1–D3 PL within the FTPR were 69.112 s, 70.484 s, and 69.919 s, respectively, whereas for the RMS delay spread within the FTPR, they were 69.740 s, 70.220 s, and 69.650 s, respectively.

These results align with expectations, as the architecture of LSTM, with its long memory, cell states, and ability to capture long-term sequential correlations, is particularly well-suited for VLC-based MBSN path loss and RMS delay data. Even though the RMSE was relatively comparable to other methods, like GRU, our design achieved low complexity, moderate parameter tuning, and faster training times compared to other models while ensuring the same standard practices. The performance of LSTM demonstrates a superior alignment with the intricate temporal dependencies in our data, solidifying its position as the most effective and reliable model for this purpose.

When implemented in real hospital settings, LSTM models face practical challenges, including energy consumption, system integration, computational complexity, and the dynamic nature of healthcare environments. To address such challenges, the authors of [68] applied LSTM to optimize Hospital Management Systems performance, analyzing historical and real-time data across two resource allocation scenarios. The model demonstrated a strong alignment between predicted and actual outcomes, with residual errors tightly around zero. In contrast, the authors of [69] used LSTM to predict patient visits at a community health center based on 43 months of historical data. The results showed that LSTM outperformed the other models, achieving a Mean Absolute Percentage Error (MAPE) of 4.714, a Mean Absolute Error (MAE) of 154.796, and an RMSE of 167.631. This indicates that the LSTM model can maintain high operational accuracy and robustness while adapting to dynamic scenarios. Furthermore, the results suggest that challenges such as computational complexity can be mitigated by the model’s ability to learn temporal patterns efficiently. These findings highlight the potential of LSTM models to overcome key challenges in real hospital environments, including reducing patient wait times, improving staff scheduling, and enhancing overall patient outcomes.

Therefore, throughout this paper, we established ML algorithms to estimate channel characteristic parameters, namely PL and RMS delay spread, in indoor VLC-based MBSNs within two hospital environments. This work contributes to the overall understanding of the IoMT and its integration into 6G networks. These findings underline the significance of ML-driven channel modeling for advancing MBSN technologies in hospital environments, paving the way for more efficient and reliable communication systems in 6G-enabled healthcare.

## 4. Conclusions

This paper introduces realistic statistical models for channel modeling in hospital environments and ML-based algorithms for adaptive modulation and channel parameter estimation in VLC-based MBSNs, considering wavelength dependency, random trajectories, and real-world hospital scenarios.

In our efforts to improve SE performance, we explored multiple modulation schemes: a Q-learning-based adaptive modulation, a KNN-based adaptive modulation, and a non-adaptive scheme that mainly serves as a reference point. The Q-learning-based modulation scheme demonstrated dynamic adaptability to changes in both the system model and the environment, all without the need for explicit CSI. Meanwhile, by balancing exploration and exploitation, the Q-learning algorithm gradually improved its SER performance until the required SER was reached. Compared to the non-adaptive approach, the KNN method demonstrated enhanced SE, though it occasionally did not satisfy the required SER. Conversely, although the Q-learning method reliably met the target SER, its SE occasionally lagged behind the optimal value due to quantization restrictions and a cautious strategy close to the desired SER. Enhancing precision is possible by increasing quantization levels, though this comes at the cost of added complexity. Future work should focus on refining the quantization process or adopting neural networks as a replacement. Moreover, other adaptive modulation algorithms can be explored to improve SE performance in VLC-based MBSNs. In addition, in environments with high data rates, where delay plays a key role in transmission, advanced RL models can be leveraged to monitor user mobility.

Beyond modulation, the study also explored channel parameter estimation for reliable VLC communication. The method used in this section was LSTM, which proved to be the best-performing ML technique. The simulation results show that in the ICU ward, D1 has the highest RMSE for path loss (1.6797 dB) and RMS delay spread (1.0567 ns). In the FTPR scenario, D3 shows the highest RMSE for path loss (1.0652 dB) and RMS delay spread (0.7657 ns). The accurate estimation of VLC channel parameters, such as DC gain and RMS delay spread, is vital for robust communication systems, with ML algorithms improving reliability and efficiency. These findings show that the performance of ML algorithms for estimating path loss and RMS delay spread in VLC-based MBSNs depends heavily on the photodetector location and scenario geometry, which are key in VLC channel modeling.

## Figures and Tables

**Table 1:** A comparison between 5G and 6G KPIs.

KPI	5G	6G
Traffic capacity	10 Mb/s/m^2^	≈1–10 Gb/s/m^3^
Data rate: downlink	20 Gb/s	1 Tb/s
Data rate: uplink	10 Gb/s	1 Tb/s
Uniform user experience	50 mb/s, 2D	10 Gb/s, 3D
Latency (radio interference)	1 ms	0.1 ms
Jitter	Not Specified	1 µs
Reliability (frame error rate)	1–10^−6^	1–10^−9^
Energy/bit	Not Specified	1 pJ/b
Localization precision	10 cm in 2D	1 cm in 3D

**Table 2:** Comparative analysis of ML-driven approaches for Link Adaptation in RF systems.

Ref.	Method	System Model	Proposed ML Model
[36]	Deep convolutional neural network (SL)	Conventionally coded MIMO-OFDM wireless system	- Establishes relationships between MCS and feature sets - Feature space: Includes SNR for each subcarrier along with noise variance - Increased complexity due to high feature dimensionality - Functions without preprocessing steps - Demands a significant dataset size for proper learning - Prior environment knowledge is required
[37]	Deep Q-learning (RL)	Indoor single-input single-output (SISO) wireless system	- Predicts current CSI and performs link adaptation using outdated CSI - State space: the most recent τ transmitted frames are utilized for RSS measurements - Action space: Several QAM modulation orders - Eliminates quantization errors - Prior environment knowledge not required
[38]	Deep Q-learning (RL)	Wireless system over Rayleigh-faded channel model	- Adaptive modulation using deep Q-network with a trial strategy - State space: Segmentation of the SNR range to establish rate regions - Action space: Utilizes Gray-coded MPSK schemes for modulation - Eliminates quantization errors - Prior environment knowledge not required
[39]	Online Deep Learning (ODL)	Massive MIMO-OFDM wireless system	- Fully connected neural network initially trained on conventional algorithm outputs and continuously fine-tuned with service feedback - Retrains online using service feedback (ACK/NACK) to adjust MCS - Feature space: Sub-band SINR for each Rx antenna, reported CQI, time since last sounding, cell RSRP, and the current MCS - Improves user throughput over classical OLLA - Prior environment knowledge not required
[40]	Latent Thompson Sampling (LTS)	Fading wireless channels as a multi-armed bandit	- Models each MCS as an arm and exploits inter-dependence between schemes. - State space: Low-dimensional latent channel-SINR distribution, inferred and updated from ACK/NACK history - Action space: Discrete MCS choices modeled as arms of the bandit - Automatically tracks channel dynamics without manual parameter tuning - Improves link throughput over classical adaptation methods - Prior environment knowledge not required

**Table 3:** Comparative analysis of ML-driven Link Adaptation approaches in AUWC systems.

Ref.	Method	System Model	Proposed ML Model
[41]	Dyna-q algorithm (RL)	Autonomous underwater vehicle (AUV)	- Predicts the current channel state and adapts modulation based on the predicted current CSI - State space: effective SNR - Action space: QPSK, 8PSK, and BPSK
[42]	Hot-booting Q-learning algorithm (RL)	Underwater acoustic	- Dynamically adjusts modulation and coding schemes to optimize QoS by evaluating multiple transmission parameters - State space: Several transmission factors of present and prior packets - Action space: MFSK and coherent single carrier modulation
[43]	Multi-layer perceptron (MLP) network (SL)	Acoustic internet of underwater things (IoUT)	- Key Challenge: Substantial propagation loss and extreme channel variations - Conventional AMC: Depends on SNR-BER correlation - Link quality parameters: SNR, BER, frequency shift, and delay spread - Demonstrated weak SNR-BER correlation in underwater channels
[44]	LSTM-enhanced DQN-based adaptive modulation (RL)	Underwater acoustic	- Key Challenge: Limited observability of the acoustic channel - Hybrid RL-LSTM architecture - Improved underwater communication model - Outdated CSI-based link adaptation - State space: Effective SNR derived from preceding time slots - Action space: 8PSK, QPSK, 16QAM, and BPSK - Eliminates quantization errors - Prior environment knowledge not required

**Table 4:** Existing ML-based VLC channel estimation studies.

Ref.	Method	System Model	Machine Learning Improvements
[48]	Extreme Learning Machine (ELM)	Underground mining based VLC system	Improved BER under harsh conditions results in performance close to perfect channel estimation case and outperforms traditional methods
[49]	Artificial Neural Network (ANN)-based ML	Industry channel conditions in a 3D VLP system	Minimize positioning errors and enhance system accuracy under the smoke channel
[50]	ML-based XGBoost	Indoor VLP system to track the smart trolley’s position	Enhanced deployment speed by reducing training time and maintaining comparable positioning accuracy
[51]	Long Short Term Memory (LSTM)	Indoor VLC channel	Superior BER performance compared to KF, which improves accuracy and system robustness
[52]	Long Short Term Memory (LSTM)	IRS-aided nonlinear VLC system	LSTM outperform traditional methods in performance
[53]	LSTM, GRU, and Sparse Autoencoders (SAEs)	Multi-wavelength VLC system with tricolor LED sources	SAEs achieves the best channel modeling performance among other ML algorithms
[54]	Hybrid DNN	Vehicular (V-VLC) and IEEE 802.11p network systems	Outperform traditional models in terms of higher detection accuracy and lower error estimation
[55]	DNN, YOLO v3, and Kalman Filter	Indoor VLC system using different modulation techniques	DNN effectively reduces BER more effectively than KF for all proposed modulation techniques
[56]	Random Fourier Features (RFF) based ML	Nonlinear VLC systems	Provides lower training complexity while improving accuracy
[57]	Federated Learning (FL)	Overview VLC networks based on various applications	Reduces data transfer cost, improve privacy and performance

**Table 5:** LSTM architecture parameters.

Parameters	Specification
Optimizer	ADAM
Number of iterations	800
Learning Rate	0.001
Number of Epochs	400
Number of Hidden units for LSTM layer	55

**Table 6:** System model and Q-Learning model parameters.

Simulation Parameters	Value
μ	{2,4,8,16,32,64}
Modulation Scheme	M-PAM
$N_{0}$	$6.464^{-23}$
Min ϵ	0.001
Max Episodes	500
σ	0.5
γ	0.5
Responsivity of PDs	1
$P_{elec}$	10 dBm
$SER_{tar}$	$10^{-3}$

**Table 7:** Estimated path loss and RMS delay within an ICU Ward through different techniques.

Technique	ICU Ward					
	**RMSE of** PL **(dB)**			**RMSE of** $\tau_{\mathrm{RMS}}$ **(ns)**		
	**D1**	**D2**	**D3**	**D1**	**D2**	**D3**
LSTM	1.6797	1.1679	1.1464	1.0567	0.9348	0.8784
GRU	1.7060	1.1808	1.1774	1.0794	0.9593	0.8840
RNN	1.7398	1.2647	1.1785	1.0904	0.9734	0.9039
SVR	1.8470	1.3671	1.2654	1.1774	0.9769	0.9107
KNN	2.3142	1.8848	1.7834	1.8088	1.5987	1.4401

**Table 8:** Estimated path loss and RMS delay within FTPR through different techniques.

Technique	FTPR					
	**RMSE of** PL **(dB)**			**RMSE of** $\tau_{\mathrm{RMS}}$ **(ns)**		
	**D1**	**D2**	**D3**	**D1**	**D2**	**D3**
LSTM	0.7210	0.7327	1.0652	0.5830	0.6230	0.7657
GRU	0.7359	0.7832	1.1480	0.6183	0.6352	0.8555
RNN	0.7663	0.7929	1.1886	0.6237	0.6509	0.8509
SVR	0.7829	0.8184	1.1762	0.6277	0.6753	0.8834
KNN	0.9110	0.9770	1.7908	0.8199	0.9602	1.2166

**Table 9:** Time complexity of an ICU ward.

Technique	ICU Ward					
	**Execution Time of** PL **(s)**			**Execution Time of** $\tau_{\mathrm{RMS}}$ **(s)**		
	**D1**	**D2**	**D3**	**D1**	**D2**	**D3**
LSTM	68.051	65.854	66.229	69.946	68.786	68.948
GRU	70.197	72.190	68.958	72.711	69.671	73.468
RNN	70.368	72.578	73.488	73.018	72.917	73.787

**Table 10:** Time complexity of FTPR.

Technique	FTPR					
	**Execution Time of** PL **(s)**			**Execution Time of** $\tau_{\mathrm{RMS}}$ **(s)**		
	**D1**	**D2**	**D3**	**D1**	**D2**	**D3**
LSTM	69.112	70.484	69.919	69.740	70.220	69.650
GRU	72.531	71.791	70.652	70.491	71.849	70.650
RNN	73.353	72.299	71.616	71.625	73.173	75.559

## Data Availability

Data are contained within the article.
